# Influence of Sex and 1,25α Dihydroxyvitamin D_3_ on SARS-CoV-2 Infection and Viral Entry

**DOI:** 10.3390/pathogens14080765

**Published:** 2025-08-02

**Authors:** Nicole Vercellino, Alessandro Ferrari, José Camilla Sammartino, Mattia Bellan, Elizabeth Iskandar, Daniele Lilleri, Rosalba Minisini

**Affiliations:** 1 Department of Translational Medicine, Università del Piemonte Orientale, 28100 Novara, Italy; nicole.vercellino@uniupo.it (N.V.); mattia.bellan@med.uniupo.it (M.B.); rosalba.minisini@med.uniupo.it (R.M.); 2 Microbiology and Virology Unit, Fondazione IRCCS Policlinico San Matteo, 27100 Pavia, Italy; alessandro.ferrari04@universitadipavia.it (A.F.); e.iskandar@smatteo.pv.it (E.I.); 3 National PhD Programme in One Health Approaches to Infectious Diseases and Life Science Research, Department of Public Health, Experimental and Forensic Medicine, University of Pavia, 27100 Pavia, Italy; 4 Department of Clinical-Surgical, Diagnostic and Pediatric Sciences, Università degli Studi di Pavia, 27100 Pavia, Italy; jose.sammartino@iusspavia.it; 5 Center for Autoimmune and Allergic Disease (CAAD), Università del Piemonte Orientale, 28100 Novara, Italy; 6 Department of Internal Medicine and Rheumatology Unit, Azienda Ospedaliero-Universitaria, Maggiore della Carità, 28100 Novara, Italy; 7 Emergency Medicine Department, Azienda Ospedaliero-Universitaria, Maggiore della Carità, 28100 Novara, Italy

**Keywords:** sex, sex hormones, estrogen, E2, 1,25α dihydroxyvitamin D_3_, calcitriol, SARS-CoV-2, COVID-19, viral entry

## Abstract

Severe Acute Respiratory Syndrome Coronavirus-2 (SARS-CoV-2) is the etiologic agent that causes the coronavirus disease (COVID-19) identified in Wuhan, in 2019. Men are more prone to developing severe manifestations than women, suggesting a possible crucial role of sex hormones. 17,β-Estradiol (E2) and 1,25 α dihydroxyvitamin D_3_ (calcitriol) act upon gene pathways as immunomodulators in several infectious respiratory diseases. In this study, we aimed to evaluate the influence of E2 and calcitriol on the VSV-based pseudovirus SARS-CoV-2 and SARS-CoV-2 infection in vitro. We infected Vero E6 cells with the recombinant VSV-based pseudovirus SARS-CoV-2 and the SARS-CoV-2 viruses according to the pre-treatment and pre–post-treatment models. The Angiotensin-Converting Enzyme 2 (ACE2) and Vitamin D Receptor (VDR) gene expression did not change under different treatments. The VSV-based pseudovirus SARS-CoV-2 infection showed a significant decrease in the focus-forming unit count in the presence of E2 and calcitriol (either alone or in combination) in the pre-treatment model, while in the pre–post-treatment model, the infection was inhibited only in the presence of E2. Th SARS-CoV-2 infection highlighted a decrease in viral titres in the presence of E2 and calcitriol only in the pre–post-treatment model. 17,β-Estradiol and calcitriol can exert an inhibitory effect on SARS-CoV-2 infections, demonstrating their protective role against viral infections.

## 1. Introduction

The outbreak of the coronavirus disease 2019 (COVID-19) was caused by the Severe Acute Respiratory Syndrome Coronavirus-2 (SARS-CoV-2). It was identified for the first time in Wuhan, China, in November 2019, and the first case was reported on the 31st of December 2019 by the World Health Organization (WHO), which declared the global pandemic on 11th of March 2020 [1]. The SARS-CoV-2 virus belongs to the Coronaviridae family as well as Severe Acute Respiratory Syndrome Coronavirus-1 (SARS-CoV) and Middle East Respiratory Syndrome (MERS-CoV), and their genus is *Betacoronavirus* included in the *Nidovirales* order [2].

The SARS-CoV-2 virus is a positive single-stranded RNA (+ssRNA) virus that is able to interact with the target cell through the binding of the glycoprotein S present on the viral envelope to the Angiotensin-Converting Enzyme 2 (ACE2) receptor. This binding mediates the fusion of the viral envelope with the host cellular membrane, a process also facilitated by a double proteolytic cleavage carried out by the transmembrane serine protease 2 (TMPRSS2) [3].

ACE2 is predominantly expressed on the surface of capillary endothelial cells, alveolar, pulmonary epithelial cells, renal epithelial cells, as well as intestinal, cerebral, and testicular cells [4]. For this reason, the SARS-CoV-2 infection is considered a multi-organ infection in humans [5] capable of exacerbating the so-called “Cytokine Storm Syndrome (CSS)” [6]. This acute condition, characterized by an uncontrolled inflammatory response and a massive, rapid release of pro-inflammatory cytokines (IL-6, Il-2, IL-7, IL-10, G-CSF, IFN-γ, IP-10, TNF-α, MCP-1, MIP-1α), involves the membrane-bound TLR-4 and the endosomal TLRs in recognizing pathogens such as the +ssRNA of SARS-CoV-2. Other TLRs induce phagocytosis and activate further molecular pathways to amplify local anti-inflammatory processes and promote the clearance of the infectious agent [6,7].

The severity and progression of the SARS-CoV-2 infection—as with many other conditions such as acute pancreatitis, rheumatoid arthritis, chronic hepatitis C, and HIV infections—are correlated with the concentration of soluble Programmed Death-Ligand 1 (sPD-L1). Indeed, PD-L1 and its receptor, Programmed Death-1 (PD-1), are considered crucial immune-regulatory markers in various pathologies [8]. They are immune checkpoint molecules involved in modulating the host immune response and regulating immune-mediated cellular damage during inflammation [9]. It has been shown that leukocytes, nonlymphoid cells, and non-hematopoietic cells—such as epithelial cells—express the transmembrane protein PD-L1 [10].

Furthermore, the literature reports that men are more prone to severe manifestations of SARS-CoV-2 infections than women [11]. This difference may result from the interaction among genetic, hormonal, and environmental factors, which seem to influence the clinical outcome of COVID-19 [12]. One hypothesis is that female hormones may play a role in the onset of COVID-19 [13].

Estrogens are involved in both female and male reproduction; they promote the development of female secondary sexual characteristics and play a key role in regulating the structure and function of the reproductive system in females [14], as well as in numerous other biological systems, including the neuroendocrine, vascular, skeletal, and immune systems [15,16]. In addition, these hormones appear to exert anti-inflammatory activity in COVID-19, with important clinical and prognostic implications. Their protective effect on endothelial cells is attributed to their antioxidative and antithrombotic properties [14]. In humans, four estrogens have been identified: Estrone (E1), 17 β-Estradiol (E2), Estriol (E3), and Estetrol (E4) [17]. Among them, E2 has the highest biological activity and can regulate cells and pathways involved in both innate and adaptive immune responses, as well as immune cell differentiation through Estrogen Receptors (ERs), ESR1 and ESR2, which encode for the nuclear receptors Erα and Erβ, respectively [16]. Upon binding its receptors, E2 can activate two distinct pathways. The slow “genomic effect” occurs when E2 forms the E2–receptor dimer complex, which enters the nucleus where it binds to estrogen response elements (EREs) [17] or to activator protein-1 (Ap1) and specificity protein-1 (Sp1) at E2-responsive gene promoters [16]. This complex regulates gene expressions by acting as a transcription factor. Moreover, under normal conditions, estrogen-mediated gene products modulate autophagy, proliferation, apoptosis, survival, differentiation, and vasodilation. On the other hand, E2 is also involved in the non-genomic signaling pathway, which is not linked to gene regulation, requiring a time span of seconds to minutes, and so it is attributed as a rapid “non-genomic effect”. E2 binds to membrane-bound ERα, Erβ, and GPER1, thereby regulating the ion channel opening or the activation of related enzymes such as Ca2+ mobilization, phosphatidylinositol 3-kinase (PI3K), and mitogen-activated protein kinase (MAPK) through the activation of nuclear transcription factors [17].

Calcitriol, the active form of vitamin D, is a steroid hormone and shares a similar structure with E2. Like E2, calcitriol plays a crucial role in immunomodulation and in fighting pathogens [16] by reducing the burden of infection through lowering viral replication rates, decreasing pro-inflammatory cytokines, and increasing concentrations of anti-inflammatory cytokines [18]. In addition, it is involved in increasing intestinal calcium absorption, regulating bone metabolism, promoting cell proliferation and differentiation, as well as exerting anti-inflammatory, antifibrotic, and antioxidant effects [19]. Calcitriol exerts most of its pleiotropic phenotypic effects inside the cell by binding to nuclear vitamin D receptors (VDRs). Once bound, VDRs are activated and dimerize with each other or with Retinoid X Receptors (RXRs) then translocate to the nucleus to engage the vitamin D receptor element (VDRE). VDREs are present on different target genes such as beta defensin, cathelicidin, and toll-like receptors, which help direct the innate immune response after the recognition of pathogenic proteins; their expression can be modulated in the presence of vitamin D [20]. Moreover, the VDR regulates the transcription of numerous genes involved in both innate and adaptive immunity, exerting modulatory effects on the immune system [21].

Based on this, our work aimed to evaluate the influence of E2 and calcitriol on the VSV-based pseudovirus SARS-CoV-2 and SARS-CoV-2 infection in vitro.

## 2. Materials and Methods

### 2.1. Cell Culture

Vero E6 cell line (ATCC, CRL-1586™) (ATCC, Manassas, VI, USA), the renal epithelial cell line of Cercopithecus aethiops, was used in order to perform all the experiments.

Cells were cultured in DMEM (1X) with GlutaMAX (Dulbecco’s Modified Eagle’s Medium) adding 10% of FBS (Fetal Bovin Serum), 1% of Streptomycin, and 1% of Penicillin. Cells were kept at 37 °C and at 5% CO_2_.

### 2.2. MTT Assay

The 3-(4,5- dimethylthiazol-2-yl)-2,5-diphenyltetrazolium bromide assay, also called MTT assay, was added in order to evaluate the toxicity of E2 and calcitriol, following the protocol instructions. Each treatment was evaluated in triplicate, and the experiment was repeated five times.

### 2.3. Treatments

The cells were treated for 15 days with E2, vitamin D3, and combination of the two compounds and DMSO (vehicle). On the 15th day, cells were frozen in 10 aliquots, 5 for the VSV-based pseudovirus SARS-CoV-2 and 5 for SARS-CoV-2 experiments, in order to ensure the use of the same batch of cells. For the infection experiments, cells were thawed and allowed to recover for 24 h before the medium was replaced with one containing the respective compounds.

### 2.4. Viruses

The VSV-based pseudovirus SARS-CoV-2 (DBA Italia S.r.l., Segrate, MI, Italia) [22], expressing the Green Fluorescent Protein (GFP) as gene reporter, was used in a set of experiments. The SARS-CoV-2 virus was originally isolated at the beginning of the Pandemic from a clinical sample at Policlinico San Matteo, Pavia. Sequence analysis of the sample revealed the presence of a specific mutation that entails an early genetic drift causing the replacement of aspartic acid with glycine at position 614 on the S protein (D614G). The infection experiments were conducted in accordance with the approved standard operating procedures of biosafety level 3 facility in the Microbiology and Virology Unit of Policlinico San Matteo, Pavia.

### 2.5. Time Course and MOI for VSV-SARS-CoV-2-S∆21

To assess the duration of infection in the experiments, a time course was performed for VSV-based pseudovirus SARS-CoV-2 using an MOI of 0.1.

### 2.6. Viral Titration Protocol for SARS-CoV-2

To evaluate the effect of E2 and calcitriol treatments on SARS-CoV-2 replication, viral titration was performed using the 50% tissue culture infectious dose (TCID_50_) method [23]. The SARS-CoV-2 isolate used for infection had been previously titrated and standardized to ensure consistent viral input across all experimental conditions. Vero E6 cells were subjected to 15-day treatment with E2, calcitriol, or their combination and then infected according to the two models described in Section 2.7 (pre-treatment and pre–post-treatment). At 24 h post-infection, culture supernatants were collected from the treated cells, centrifuged to remove debris, and used for titration. Logarithmic dilutions of each viral supernatant were incubated with naïve Vero E6 cells (3 × 10^4^ cells/well) in 96-well plates, with six technical replicates per dilution. After 72 h of incubation at 33 °C in 5% CO_2_, cells were examined for the presence of cytopathic effect (CPE). The viral titre was calculated and expressed as TCID_50_/mL using the Reed–Muench method [24]. Each experimental condition was independently repeated three times (biological replicates).

### 2.7. Models of Infection

Two different models of infection were evaluated in the presence of the compounds under study:

- Pre-treatment model: Vero E6 cells were pre-treated with the specific hormone, seeded in 96-wells plate, and the day after the treatment was removed, the virus was added in presence of the compounds; after 3 h of viral adsorption, the viral inoculum was removed, cells were washed, and then medium without treatments was added.
- Pre–post-treatment model: Infection was performed as in pre-treatment model, but after the 3 h of viral adsorption, medium with the different treatments was added.

In both models, viral titration (TCID_50_/mL) was performed using the supernatants collected 24 h post-infection from the treated cells, in order to evaluate the amount of infectious virus produced.

### 2.8. Real Time qPCR

The RNA extraction was performed using the TRIzol (TRI Reagent^®^) (Sigma-Aldrich^®^, Saint Louisan, MO, USA), according to the manufacturer’s instructions. RNA was retrotranscribed into cDNA using the High-Capacity cDNA Reverse Transcription Kit (Applied Biosystems™, Foster City, CA, USA), according to the manufacturer’s instructions.

Real Time qPCR was performed using the PowerTrack™ SYBR Green Master Mix, according to the manufacturer’s protocol. Genes were amplified using the following primers: ACE2 fw 5′-TCCATTGGTCTTCTGTCACCCG-3′, ACE2 rv 3′-AGACCATCCACCTCCACTTCTC-5′, VDR fw 5′-CGCATCATTGCCATACTGCTGG-3′, VDR rv 3′-CCACCATCATTCACACGAACTGG-5′, PD-L1 fw 5′-TGCCGACTACAAGCGAATTACTG-3′, and PD-L1 rv 3′-CTGCTTGTCCAGATGACTTCGG-5′.

### 2.9. Bio-Plex Multiplex System

Bio-PlexPro™ human cytokines screening panel (27-Plex, #M500KCAF0Y, Bio-Rad, Hercules, CA, USA) was used to perform the quantification of cytokines, chemokines, and growth factors present in the supernatant collected from infected and non-infected cells.

All sample processing was carried out according to the manufacturer’s instructions provided in the Bio-PlexPro™ Assay manual.

### 2.10. Statistical Analysis

Statistical analysis of the data and the creation of the graphs were performed using GraphPad Prism 9.4.0 (GraphPad Software, LLC, San Diego, CA, USA). Comparison of independent treatment influence on focus-forming unit (FFU) count and on receptor gene expression was performed through Mann–Whitney test. Statistically significant level was set at *p* < 0.05. Prism ranges of *p* values are indicated with asterisks: * *p* < 0.05; ** *p* < 0.01; *** *p* < 0.001; and **** *p* < 0.0001.

## 3. Results

### 3.1. MTT E2 and Calcitriol

The influence of the E2 hormone and calcitriol on cellular viability was evaluated using the MTT assay. For the control group, either DMSO or DMEM was used.

**Figure 1:**
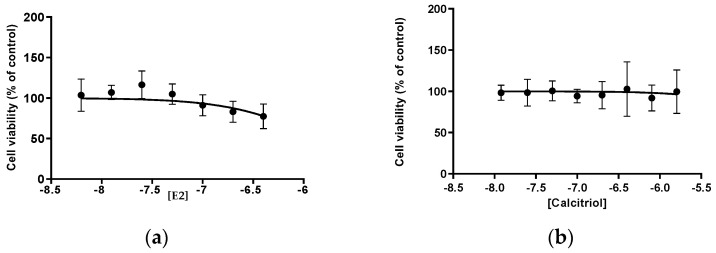
The analysis of the results of the MTT assay: the concentration of the E2 is shown on the x-axis in panel (**a**), and the calcitriol in panel (**b**) is expressed on the semi-logarithmic scale, while the y-axis represents the percentage of cell viability normalized to the control.

The viability of Vero E6 cells treated with E2 was consistently above 77% (IC50 1.398 μM), while for calcitriol it remained above 71% (IC50 42.5 μM) (Figure 1).

Based on the MTT, the concentrations of the compounds used to treat the Vero E6 cell line for 15 days were 100 nM for E2 and 100 nM for calcitriol because they revealed a minor toxic effect.

### 3.2. VSV-Based Pseudovirus SARS-CoV-2 Infection: MOI and Time Course

**Figure 2:**
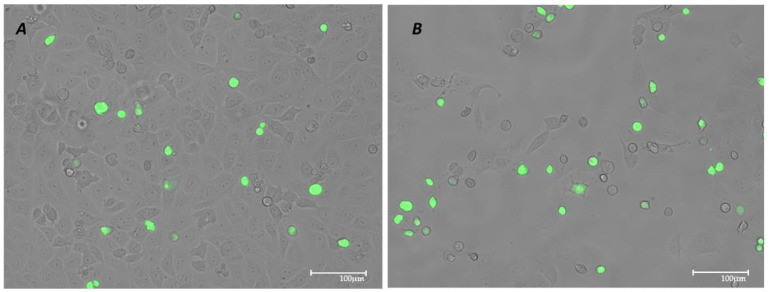
Vero E6 cell line infected with VSV-based pseudovirus SARS-CoV-2 expressing GFP (MOI 0.1) after 12 h (**A**) and 24 h (**B**) of infection visualized through fluorescence microscope. Images were acquired with digital microscope EVOS FLOID (Life Technologies), zoom 100%.

Vero E6 cells infected with the VSV-based pseudovirus SARS-CoV-2 at an MOI of 0.1 for 24 h showed a higher cytopathic effect (CPE) and cellular suffering (B) compared to 12 h of infection under the same conditions (A). Therefore, the combination of an MOI of 0.1 and a 12 h infection period was selected for subsequent experiments (Figure 2).

### 3.3. Models of Infection and Plaque Assay: VSV-Based Pseudovirus SARS-CoV-2

**Figure 3:**
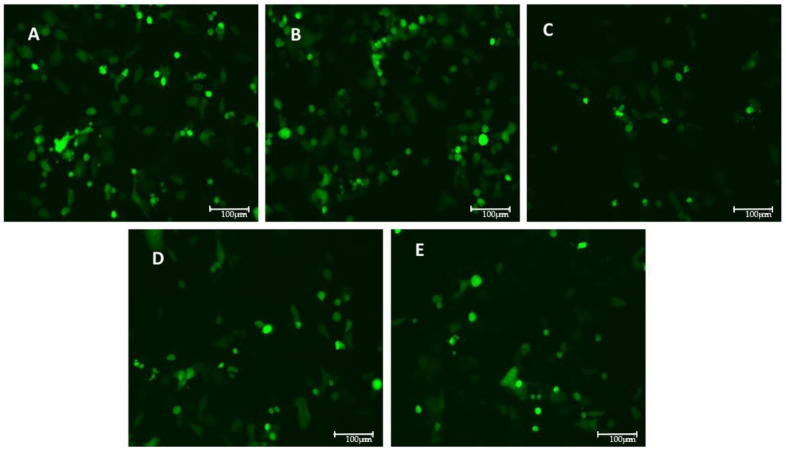
GFP-expressing VSV-based pseudovirus SARS-CoV-2 infection with MOI 0.1 for 12 h in pre-treated setting with different compounds, in particular (**A**) control (**B**), DMSO at 1:40,000 dilution (vehicle) (**C**), E2 at 100 nM (**D**), calcitriol at 100 nM, and (**E**) combination of E2 and calcitriol. Images were acquired with digital microscope EVOS FLOID (Life Technologies), zoom 100%.

**Figure 4:**
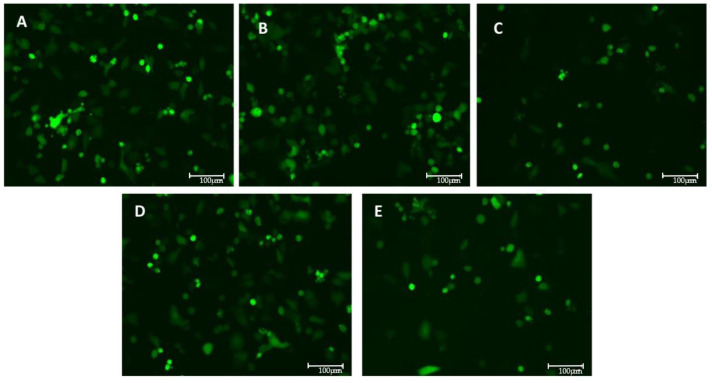
GFP-expressing VSV-based pseudovirus SARS-CoV-2 viral infection with MOI 0.1 for 12 h in pre-and post-treated setting with different compounds: (**A**) control (**B**), DMSO at 1:40,000 dilution (vehicle) (**C**), E2 at 100 nM (**D**), calcitriol at 100 nM, and (**E**) combination of E2 and calcitriol. Images were acquired with digital microscope EVOS FLOID (Life Technologies), zoom 100%.

The effects of the compounds on the viral entry were evaluated through plaque assays in two settings: pre-treatment and pre- and post-treatment. Both conditions showed a reduction in the FFU in the presence of the E2, as reported in Table A1 in Appendix A and illustrated in Figure 3 and Figure 4.

**Figure 5:**
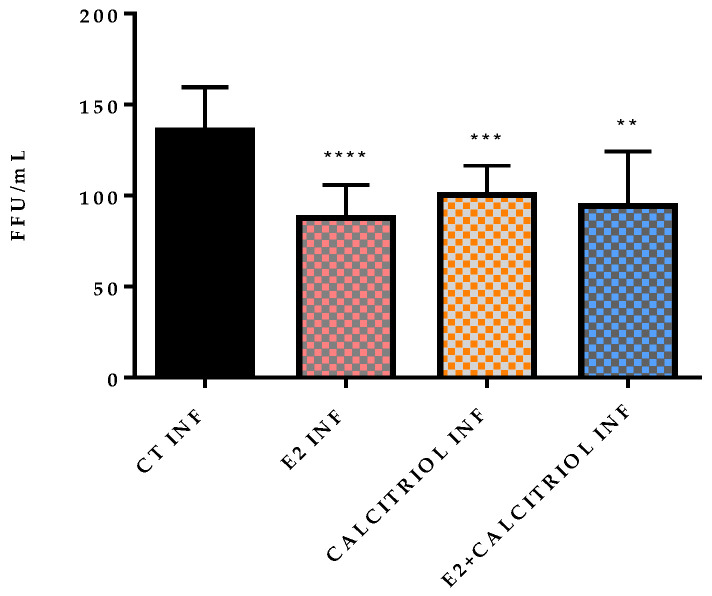
FFU/mL count after 12 h of infection with VSV-based pseudovirus SARS-CoV-2 at MOI 0.1 in presence of different compounds in pre-treatment model (E2 100 nM, calcitriol 100 nM, and same concentration in combination). *p*-value cut-off 0.05. ** *p* < 0.01; *** *p* < 0.001; **** *p* < 0.0001.

**Figure 6:**
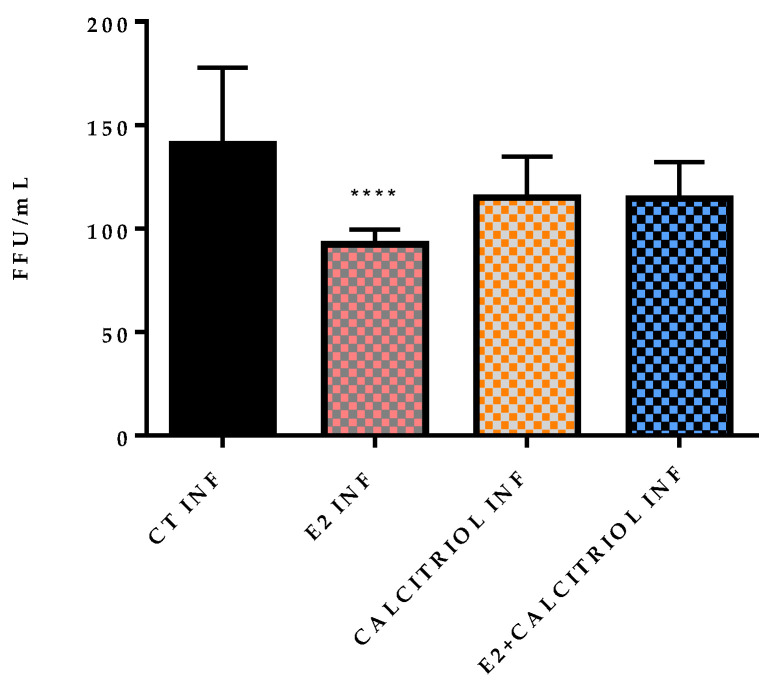
FFU/mL count after 12 h of infection VSV-based pseudovirus SARS-CoV-2 at MOI 0.1 in presence of different compounds in pre- and post-treatment model (E2 100 nM, calcitriol 100 nM, and same concentration in combination). *p*-value cut-off 0.05. **** *p* < 0.0001.

A significant decrease in the FFU count is observed in the presence of E2, calcitriol, and when the two compounds are combined in the pre-treatment condition (Figure 5). On the contrary, in the pre-and post-treatment set of experiments, a significant decrease in the FFU is obtained only in the presence of the E2 treatment alone (Figure 6). These observations are supported by the raw FFU values reported in Table A1, where a consistent reduction in viral foci is seen across biological replicates for E2, calcitriol, and their combination in the pre-treatment model.

### 3.4. Models of Infection and Viral Titre (TCID50/mL) SARS-CoV-2

**Figure 7:**
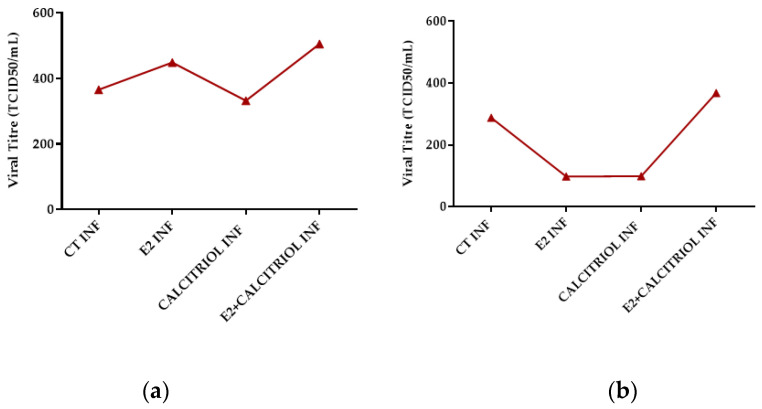
SARS-CoV-2 titre after 24 h of infection in presence of different compounds in pre- (**a**) and pre–post-(**b**) treatment models (E2 100 nM, calcitriol 100 nM, and same concentration in combination). Although not statistically significant, we observed reduction in viral titre with E2 and calcitriol alone in pre–post-treatment model, while their combination was associated with trend toward increased viral production.

On one hand, the infection with SARS-CoV-2 in the pre-treatment model revealed an inhibitory effect only in the presence of calcitriol alone. On the other hand, in the pre–post-treatment model, both E2 and calcitriol alone determined a reduction in the viral titre, while the treatment with their combination induced a slightly increased viral production (Figure 7a,b).

### 3.5. VSV-Based Pseudovirus SARS-CoV-2 and SARS-CoV-2 Entry: Gene Expression of ACE2 and VDR

**Figure 8:**
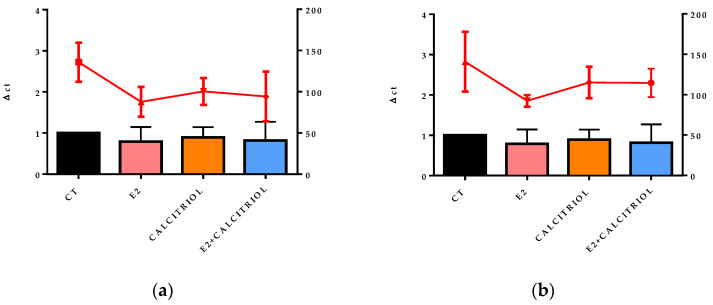
The graph shows the ACE2 gene expression (left y axis) at the baseline (t0) in the presence of the different compounds under study, and the FFU/mL count is shown on the right y axis (red line) after 12 h of infection with the VSV-based pseudovirus SARS-CoV-2 at an MOI of 0.1 in pre-treatment (**a**) and pre–post-treatment (**b**) models.

**Figure 9:**
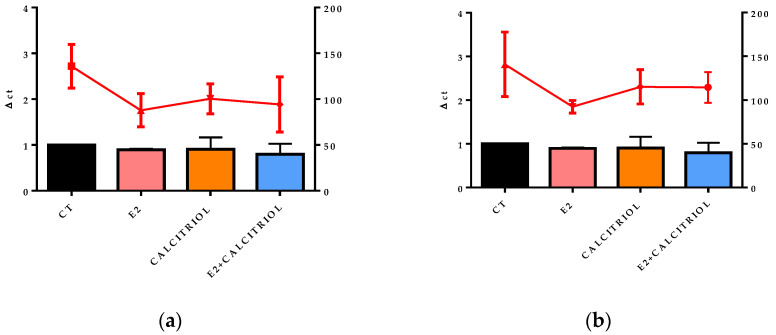
The graph shows the VDR gene expression (left y axis) at the baseline (t0) in the presence of the different compounds under study, and the FFU/mL is shown on the right y axis (red line) after 12 h of infection with the VSV-based pseudovirus SARS-CoV-2 at an MOI of 0.1 in pre-treatment (**a**) and pre–post-treatment (**b**) models.

**Figure 10:**
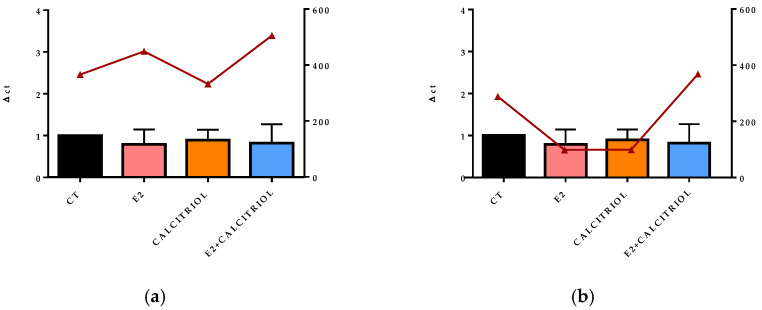
The graph shows the ACE2 gene expression (left y axis) at the baseline (t0) in the presence of the different compounds under study, and the viral titre SARS-CoV-2 (expressed in TCID 50/mL) after 24 h of infection in pre-treatment (**a**) and pre–post-treatment (**b**) models is shown on the right y axis (red line).

**Figure 11:**
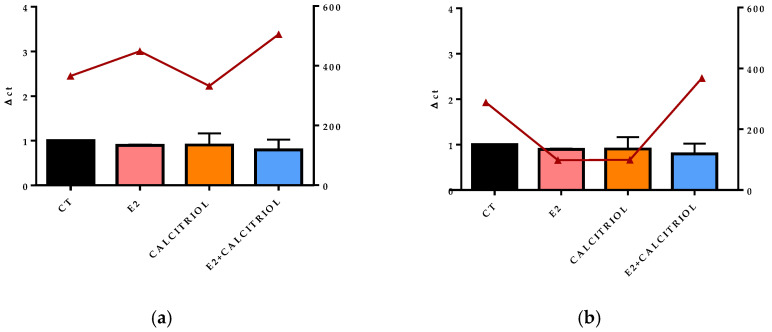
The graph shows the VDR gene expression (left y axis) at the baseline (t0) in the presence of the different compounds under study, and the viral titre of SARS-CoV-2 (expressed in TCID 50/mL) after 24 h of infection in pre-treatment (**a**) and pre–post-treatment (**b**) models is shown on the right y axis (red line).

The gene expression of the ACE2 receptor located on the cellular membrane and the nuclear VDR was evaluated at the baseline (t0), corresponding to the time of the viral inoculation. No significant differences in the expression levels of either receptor were observed across the different treatment conditions, as shown in Figure 8a,b, Figure 9a,b, Figure 10a,b and Figure 11a,b.

**Figure A1:**
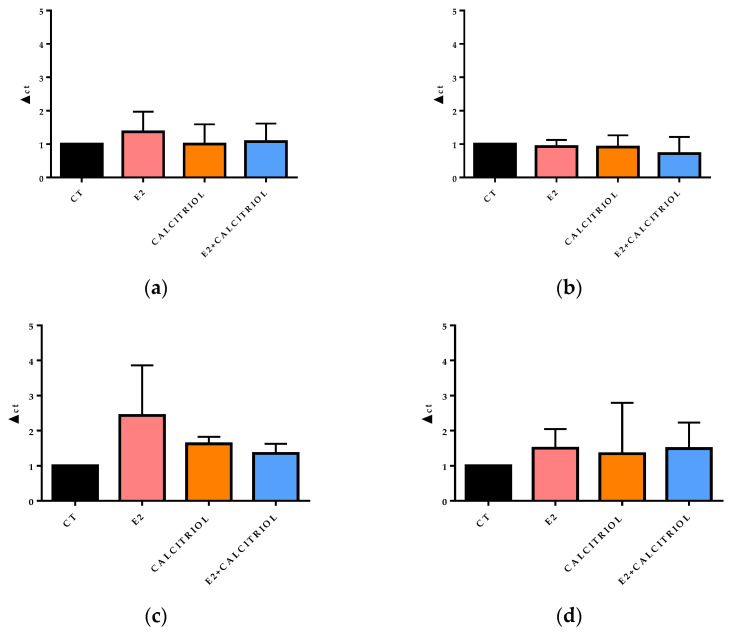
The two sets of graphs above show the ACE2 (**a**,**b**) and VDR (**c**,**d**) gene expression on Vero E6 cells also evaluated on non-infected cells at 12 h (mock-infected cells).

**Figure A2:**
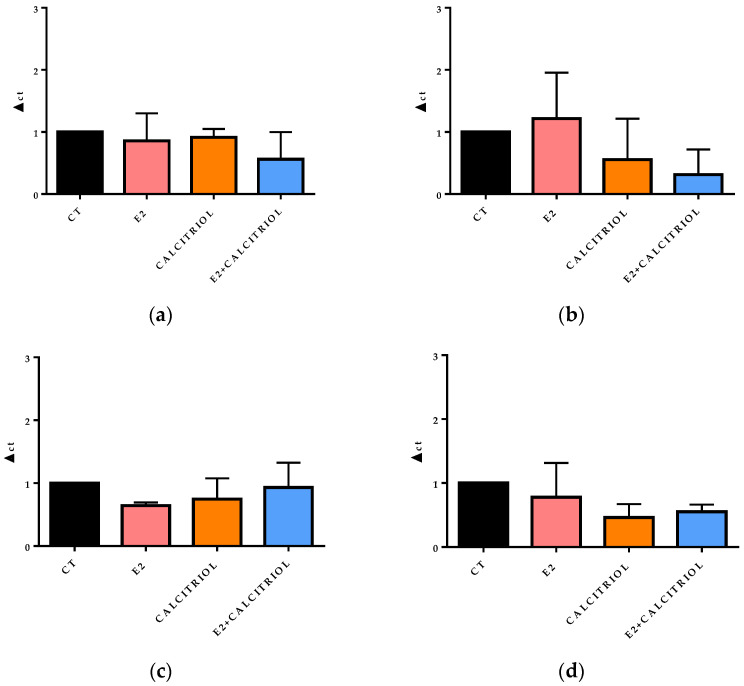
The two sets of graphs above show the ACE2 (**a**,**b**), and VDR (**c**,**d**) gene expression on Vero E6 cells also evaluated on non-infected cells at 24 h (mock-infected cells).

The ACE2 and VDR gene expression on Vero E6 cells has been evaluated on non-infected cells also at 12 and 24 h after the mock-infected cells. No significant differences in gene expression were observed (Figure A1 and Figure A2 in Appendix A).

### 3.6. Cytokines

**Figure 12:**
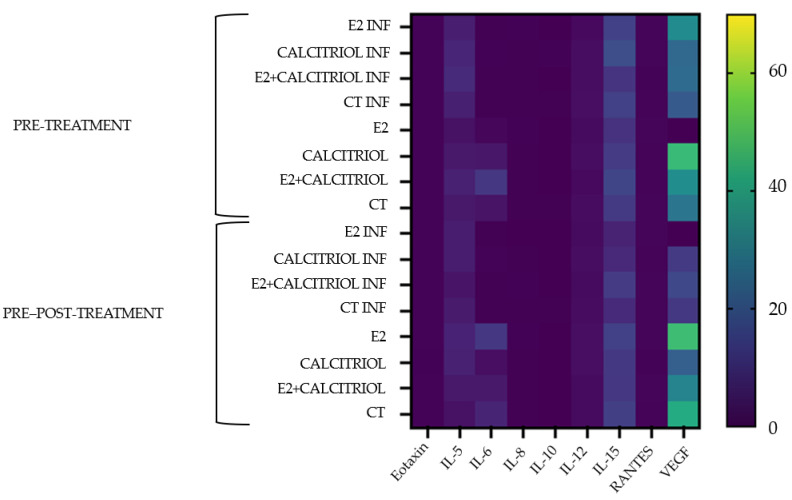
A heat map showing the increase or decrease in cytokine levels in the supernatant of Vero E6 cells infected by SARS-CoV-2 compared to non-infected ones. Each column of the heat map represents different cytokines, and rows indicate the different treatments in the two models: the pre-treatment and pre–post-treatment. The blue–yellow color code represents low-to-high average cytokine/chemokine/growth factors levels.

The levels of cytokines present in the supernatant of both Vero E6 cells infected and not infected with SARS-CoV-2 were determined, and the results are shown in Figure 12. The average concentration values of detectable cytokines and growth factors are reported in Table A2 (in Appendix A).

In the heat map, only the measurable cytokine/chemokine/growth factors were reported: Eotaxin, IL-5, IL-6, IL-8, IL-10, IL-12, IL-15, RANTES, and VEGF. As shown in Figure 12, only the VEGF resulted in an increment in the concentration.

### 3.7. SARS-CoV-2 Infection and PD-L1 Expression

**Figure 13:**
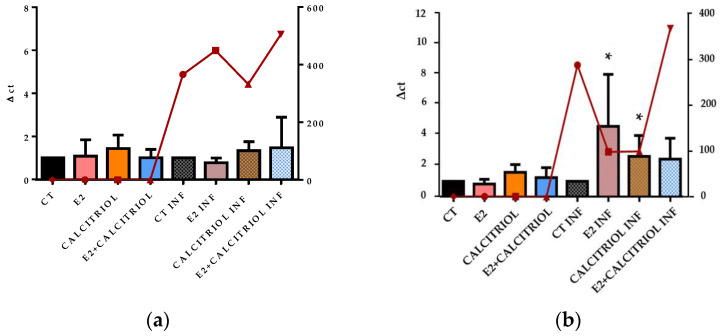
The PD-L1 gene expression in pre-treatment (**a**) and pre–post-treatment (**b**) models in the presence and absence of a SARS-CoV-2 infection in the Vero E6 cell line. * *p* < 0.05.

The level of the PD-L1 expression was evaluated after the SARS-CoV-2 infection in the presence of the different treatments and in the two models. An increase in the PD-L1 gene expression level was found in the pre–post-treatment model under the influence of E2, calcitriol, and their combination (Figure 13).

## 4. Discussion

Over the past two decades, three sub-species of Beta strain coronaviruses, MERS-CoV, SARS-CoV, and most recently SARS-CoV-2, have emerged, initially as zoonotic infections and later through human-to-human transmission. These viruses are characterized by high pathogenicity and large-scale diffusion [25]. SARS-CoV-2 is associated with a range of clinical manifestations, from asymptomatic to life-threatening conditions [25]. Among individuals, the response to systemic inflammation appears to be influenced by both viral features and host factors, such as comorbidities, age, and gender (including sex steroid hormones), which may play a crucial role [11]. It is known that men have a higher risk of death compared to women [26], even though the incidence of SARS-CoV-2 infection seems to be similar between the two sexual groups [25]. Among steroid hormones, calcitriol is widely known not only for its role in modulating the host immune response against various intracellular pathogens—such as *Mycobacterium tuberculosis* and other infectious agents—but also for its anti-inflammatory properties [27].

In our previous work (unpublished), we tested the effect of different sexual hormones, E2, progesterone, and testosterone, on cells treated for 18 h (overnight, ON), and we did not observe an inhibitory effect of E2 on the viral entry. At the same time, Lemes et al. (2021) reported a reduction in the viral load 24 h post-infection in the presence of E2 and that the ACE2 receptor gene expression, in mock Vero E6 cells, was not altered during a prolonged exposure to E2 (48 h) [13]. Moreover, some authors have suggested that a synergy between calcitriol and estrogen may contribute to sex-related differences in the outcome of patients with COVID-19 [28], while Mok et al. (2023) showed an inhibitory effect of calcitriol on the viral load. Therefore, in light of the existing literature [27], we set out to re-evaluate our data by extending the treatment duration and adding the treatment with calcitriol.

The result of the cell viability assays (MTT) on the Vero E6 cell line suggested that the optimal concentration of calcitriol was 100 nM, at which the cell viability consistently remained above 71%. This concentration was therefore used in all subsequent experiments. For E2 it was also 100 nM, with a cell viability consistently above 77%. This assay also allows us to clarify that during the infection, the decrease in SARS-CoV-2 titres was not due to the cytotoxic effects of the compounds under study. In particular, the concentrations for E2 and calcitriol were selected to fall within the physiological range in order to ensure that the in vitro results could be meaningfully translated to in vivo models.

Based on these findings—and considering that the impact of sex hormones in viral infections has become increasingly evident over the years—the present study aimed to evaluate the influence of E2 and calcitriol on the VSV-based pseudovirus SARS-CoV-2 and SARS-CoV- 2 infection in vitro. According to our data, both E2 and calcitriol reduced the viral infection when tested individually. However, when used in combination, there was no additional reduction in infection with the recombinant virus; on the contrary, the combination seemed to favor infections with SARS-CoV-2.

After 15 days of treatment, the gene expression of ACE2 and VDRs performed at the baseline (t0) on the non-infected controls underlined how the prolonged treatment with E2, calcitriol, or their combination did not influence the expression levels of these genes. Nevertheless, the literature presents opposing results with reports of both enhanced and decreased expressions of ACE2 and VDR after shorter exposure periods to the different compounds [27,28]. By analyzing the expression of these receptors at the t0, in the moment at which the virus enters the cell and starts to replicate in the presence of different treatments, this allows us to observe that the estrogen inhibitory effect is not related to the ACE2 receptor modulation. Similarly, there was no difference in the expression of these two receptors at 12 h and 24 h in mock-infected cells.

We found a significant reduction in the FFU count in the pre-treatment set in the presence of E2, calcitriol, and their combination. In contrast, in the pre–post-treatment, a significant decrease was observed only with E2 in the plaque assay. In this model, the observed reduction in the viral FFU following the E2 treatment could reflect the activation of a TMPRSS2-independent mechanism, possibly related to the altered endosomal trafficking or membrane fusion, as E2 has been reported to downregulate the expression of TMPRSS2 [13]. Given that SARS-CoV-2 can rely on endosomal cathepsin-dependent pathways for viral entry [29], the continuous exposure to E2 may affect these intracellular processes. Estrogens, indeed, are known to influence the vesicle trafficking, pH regulation [30], and membrane fusion events, which may interfere with the pseudovirus entry.

Based on our previous data (unpublished), where the cells underwent an ON treatment with the hormones, and considering the gene expression results, we did not anticipate the inhibitory effect observed in the current study. Notably the main difference from our previous work (unpublished data) is the treatment duration. During the ON treatment, neither the pre-treated nor the pre–post-treated models showed a significant inhibitory effect of E2 on the VSV-based pseudovirus SARS-CoV-2 viral entry. However, with the current E2 treatment for 15 days, which more closely reflects physiological conditions in the female body, we obtained a significant inhibitory effect. This estrogen-mediated effect was not enhanced by the addition of calcitriol, although when calcitriol was used alone, it was able to induce a reduction in the viral FFU. Indeed, when the calcitriol–VDR–RXR complex translocates inside the nucleus in order to bind the VDRE, it is able to induce the activation of both co-activators and co-repressors to regulate different genes. Of particular interest is the antimicrobial peptide cathelicidin (LL-37), which is able to inhibit cathepsin L, an enzyme involved in the early stages of the SARS-CoV membrane fusion and viral entry into the host cell through a dual mechanism: by an association to the receptor binding domain of S1 and during the so-called “spike blocking and ACE2 cloaking” process, which diminishes the recruitment of ACE2 [27].

The same viral infection models were applied in the set of experiments performed on Vero E6 cells infected with the clinical SARS-CoV-2 variant. To maintain reproducibility, cells treated for 15 days were frozen in multiple aliquots to be used in both experiments. In the pre-treated model, no significant effect of the treatments on the infection was observed. However, more interesting results were obtained in the pre–post-treatment model setting, in which both E2 and calcitriol alone seemed to counteract the viral infection, which is consistent with our findings in the experiments performed with the recombinant virus. Specifically, we observed a decreased viral production in the presence of the two hormones separately but an unexpected increase in the combination setting was observed. It is important to note that the viral titre measured by TCID_50_ reflects the infectious particles released into the supernatant by treated and infected cells, rather than the primary infection of treated cells per se. Additionally, both compounds belong to the family of steroid hormones that are characterized by binding to their specific nuclear receptor (Erα and VDR) and to more generic rapid-response steroid-binding receptors (non-genomic pathway). These pathways may interact antagonistically when both hormones are administered together [31]. Therefore, it is plausible that E2 and calcitriol, when administered in combination, can interfere with each other’s signaling pathways at the transcriptional or epigenetic level, potentially blocking their individual antiviral effects or even activating unforeseen protective genes for the virus. This antagonistic interaction could result in a non-synergistic modulation of host responses, reducing the host’s ability to recognize and neutralize the virus, especially in the early stages of infection. Furthermore, calcitriol primarily exerts its antiviral activity by modulating immune responses and activating interferon pathways [30]. However, Vero E6 cells lack a functional interferon system [32], which likely limits calcitriol’s antiviral effectiveness in this model. The combined treatment with calcitriol and E2 may also disrupt the intracellular signaling or receptor activity required for E2’s protective effect, possibly through receptor crosstalk or competition for co-regulatory factors. As previously mentioned, the VSV-based pseudovirus SARS-CoV-2 represents a safe and efficient model to study the viral entry mediated by the S protein, but it lacks several crucial features of a fully replicating virus. Moreover, the pseudovirus only mimics the entry phase and does not replicate or express other viral structural proteins such as N, M, or E, which play essential roles in modulating host cellular and immune responses. Therefore, it cannot assess the potential combined effects of E2 and calcitriol on stages beyond viral entry, such as replication or immune evasion. As a result, the slight increase in the infection observed in this model under the combined treatment likely reflects partial intracellular interactions and may not necessarily translate to an enhanced viral infection in physiological contexts. In contrast, the second setting using the SARS-CoV-2 D614G variant allowed the analysis of the full viral life cycle. A fully competent SARS-CoV-2 virus engages the host machinery throughout its entire replication cycle and is able to activate innate immune pathways, including stress responses and inflammatory signaling. Mok et al. (2023) reported some controversial SARS-CoV-2 infection results: while the antimicrobial peptide LL-37—induced by calcitriol—can inhibit cathepsin L, a key player in viral entry, the SARS-CoV-2 infection may also impair vitamin D signaling, reducing the effectiveness of endogenous calcitriol in Vero E6 cells [27]. Our results are further supported by evidence showing that calcitriol primarily targets post-entry phases of the SARS-CoV-2 infection during the primary infection. The LL-37 upregulation as a result of the calcitriol treatment may help prevent secondary infections and reduce the overall viral titre [27]. Additionally, the literature reports that among the 0.01, 0.1, 1, 10, and 20 µM calcitriol treatments on Vero E6 cells for 48 h, the 20 µM was the dose that significantly reduced the infection in Vero E6 cells [27]. However, that concentration exceeds the physiological range; therefore, in our study, we used a 100 nM concentration, which aligns with physiological levels and may explain the differences in observed antiviral effects.

In contrast to in vitro findings, in vivo studies have shown that calcitriol does not exert significant protective effects against SARS-CoV-2 infections: K18-hACE2 mice pre- and post-treated with calcitriol did not demonstrate the protection from the SARS-CoV-2 infection [27]. Moreover, these results suggest that the usual therapeutic dosage of the active form of vitamin D may be ineffective in preventing infection or improving the prognosis in COVID-19 patients. Increasing the dosage, furthermore, is associated with potential side effects, such as hypercalcemia [27].

Finally, the measurement of the cytokines in the supernatant of the SARS-CoV-2 infected and non-infected cells through the BioPlex assay (Bio-Plex manager software 6.0) suggested that only a few cytokines are influenced by the infection on this cell line. Among the twenty-seven cytokines/chemokines/growth factors present in the assay belonging to the main common molecular signaling pathways associated with inflammation, a detectable value was observed only for Eotaxin, IL-5, IL-6, IL-8, IL-10, IL-12, IL-15, and RANTES. However, no significant differences were observed in their concentrations between infected and non-infected conditions or among the various treatment groups. As expected, interferons were not detected in these cells as reported in the literature [33]. Interestingly, the assay revealed an increased concentration of the vascular endothelial growth factor (VEGF). Elevated VEGF levels during SARS-CoV-2 infections have been associated with a hyperinflammatory state that contributes to cytokine storm development. The dysregulation of the VEGF is also implicated in the pathogenesis of severe COVID-19, particularly in cases associated with acute respiratory distress syndrome (ARDS), pulmonary edema, systemic viral dissemination, endothelial dysfunction, thrombosis [34], and immune cell extravasation [35]. Recent data in the literature have shown that VEGF levels in a patient’s sample are higher in COVID-19 patients than in healthy subjects, and its level is correlated with the severity of the disease [35]. Regarding the VEGF, Mura et al. observed a controversial role of the VEGF in ARDS development; indeed, increased levels of the VEGF are reported during the lung recovery phase involving neoangiogenesis, membrane repair, and the clearance of the pulmonary edema [36]

When a virus is in contact with the host epithelial cell, it leads to PD-L1 expression on that cell, and an increased expression of PD-L1 on the cells of innate immunity in COVID-19 patients could be driven by both SARS-CoV-2 and/or the inflammatory surrounding environment created by the infection itself. “Transiently” targeting this axis can produce a beneficial result to fight the viral infection [10]. On this basis, we decided to analyze the level of the gene expression of this molecule in the context of SARS-CoV-2 infections. Our results showed that calcitriol increases the PD-L1 expression, particularly in infected cells, supporting its role as an immune modulator. This finding aligns with the work of Eduarda et al. (2022), who demonstrated that calcitriol can help balance the PD-1/PD-L1 axis and reduce inflammation [8,16].

Additionally, elevated levels of soluble PD-L1 (sPD-L1) may suppress the T cell activation by binding to PD-1 receptors on their surface, initiating a positive feedback loop that further increases the PD-1 expression [8].

We acknowledge some limitations in our study. Vero E6 cells, due to their non-human origin and lack of TMPRSS2 protease expression, are not the ideal model for studying all aspects of the SARS-CoV-2 infection. Nevertheless, this cell line is widely used for the isolation, propagation, and high-titre study of SARS-CoV-like viruses [37]. In the future, we intend to validate the current results on human cell lines such as Calu-3, primary human bronchial epithelial cells (HBECs), or A549 cells overexpressing ACE2 in order to have a comprehensive understanding of the mechanisms involved in SARS-CoV-2 infections. Moreover, we will perform a viral RNA quantification at different times post-infection in order to better understand which stage of the viral replication cycle is affected by the treatments in the two models of infection.

In conclusion, our results suggest that the E2 and calcitriol treatment in vitro can exert inhibitory effects on the SARS-CoV-2 infection. Notably, E2 demonstrated a protective role, highlighting potential sex-based differences in the clinical evolution of COVID-19 infections. These differences may be explained by variations in immunological responses influenced by sex hormones. Many immune-related genes are located on the X chromosome, giving women a potential immunological advantage through a stronger innate and adaptive immune response compared to men, including enhanced Th2 responses and reduced Th1 activity [26]. As for calcitriol, while it showed some antiviral potential, further research is needed to determine the optimal dosing and conditions for its effective use.

## Data Availability

All data generated or analyzed during this study are included in this published article.

## Abbreviations

The following abbreviations are used in this manuscript:

SARS-CoV-2	Severe Acute Respiratory Syndrome Coronavirus-2
COVID-19	Coronavirus Disease 19
E2	17,β-Estradiol
Calcitriol	1,25 α Dihydroxyvitamin D_3_
ACE2	Angiotensin-Converting Enzyme 2
VDR	Vitamin D Receptor
PD-L1	Programmed Death-Ligand 1
WHO	World Health Organization
SARS-CoV	Severe Acute Respiratory Syndrome Coronavirus-1
MERS-CoV	Middle East Respiratory Syndrome
+ssRNA	Positive Single-Stranded RNA
TMPRSS2	Transmembrane Serine Protease 2
CSS	Cytokine Storm Syndrome
sPD-L1	Soluble Programmed Death-Ligand 1
PD-1	Programmed Death-1
TGFβ	Transforming Growth Factor-β
NO	Nitric Oxide
CVDs	Cardiovascular Disease
E1	Estrone
E3	Estriol
E4	Estetrol
ERs	Estrogen Receptors
EREs	Estrogen Response Elements
Ap1	Activator Protein-1
Sp1	Specificity Protein-1
PI3K	Phosphatidylinositol 3-Kinase
MAPK	Mitogen-Activated Protein Kinase
RXRs	Retinoid X Receptors
VDRE	Vitamin D Receptor Element
DMEM	Dulbecco’s Modified Eagle’s Medium
FBS	Fetal Bovin Serum
MTT	3-(4,5-Dimethylthiazol-2-yl)-2,5-Diphenyltetrazolium Bromide
VSV	Vesicular Stomatitis Virus
GFP	Green Fluorescent Protein
FFU	Foci Forming Unit
CPE	Cytopathic Effect
TCID 50	50% Tissue Culture Infectious Dose
TEM	Transmission Electronic Microscope
ON	Overnight
LL-37	Antimicrobial Peptide Cathelicidin
VEGF	Vascular Endothelial Growth Factor
ARDS	Acute Respiratory Distress Syndrome

## Appendix A

**Table A1:** The FFU count in the pre-treatment and pre–post-treatment after 12 h of infection with VSV-SARS-CoV-2-S∆21.

	Treatment			
**Pre-Treatment**				
	**CT INF**	**E2 INF**	**CALCITRIOL INF**	**E2 + CALCITRIOL INF**
	130	105	117	108
	158	95	107	145
	313	101	110	122
	137	118	125	97
	130	70	103	65
	114	70	98	54
	138	87	81	112
	130	77	83	75
	151	67	80	70
**Pre–Post-Treatment**				
	146	84	133	128
	194	97	121	126
	131	90	115	127
	114	94	94	115
	118	108	128	104
	115	90	142	130
	180	84	119	102
	119	94	80	110
	153	90	104	77

**Table A2:** This table shows the average concentration values of detectable cytokines and growth factors.

	Cytokines/Chemokines and Growth Factors Average Concentrations (pg/mL)								
	Eotoxin	IL-5	IL-6	IL-8	IL-10	IL-12	IL-15	Rantes	VEGF
**Pre-Treatment**									
E2 INF	0.34	5.6	0.07	0.23	0.00	1.43	13.04	0.57	33.36
CALCITRIOL INF	0.40	7.01	0.17	0.12	0.26	2.09	16.38	0.68	23.08
E2 + CALCITRIOL INF	0.38	8.16	0.08	0.05	0.00	2.09	10.40	0.32	24.05
CT INF	0.36	5.84	0.07	0.10	0.05	2.26	12.98	0.5	19.31
E2	0.34	3.00	0.96	0.26	0.00	1.76	9.87	0.57	0.00
CALCITRIOL	0.38	4.45	4.11	0.13	0.00	2.09	12.07	0.45	47.25
E2 + CALCITRIOL	0.38	6.16	10.95	0.05	0.00	1.43	14.02	0.44	33.87
CT	0.37	4.44	3.32	0.12	0.02	1.93	11.72	0.62	27.00
**Pre–Post-Treatment**									
E2 INF	0.34	5.3	0.13	0.00	0.00	1.76	6.56	0.57	0.00
CALCITRIOL INF	0.38	5.31	0.39	0.05	0.00	2.09	7.75	0.45	11.65
E2 + CALCITRIOL INF	0.38	3.54	0.08	0.17	0.00	1.76	12.07	0.62	14.93
CT INF	0.37	4.72	0.05	0.08	0.02	2.01	8.14	0.59	11.05
E2	0.41	6.44	10.98	0.18	0.00	2.42	13.11	0.57	48.09
CALCITRIOL	0.27	6.17	2.22	0.12	0.00	2.42	11.03	0.38	20.66
E2 + CALCITRIOL	0.38	4.16	4.44	0.05	0.00	1.76	10.91	0.57	31.32
CT	0.39	2.97	6.62	0.11	0.00	1.84	12.74	0.50	42.78
